# Aggressive Angiomyxoma of the Bladder Neck Requiring Local Excision and Mitrofanoff Formation

**DOI:** 10.1155/2015/819243

**Published:** 2015-10-28

**Authors:** M. Davari, B. W. Lamb, S. Chowdhury, C. Jameson, J. D. Kelly, T. Greenwell

**Affiliations:** ^1^University College London Hospitals Medical School, London WC1E 6BT, UK; ^2^Department of Urology, University College London Hospitals NHS Foundation Trust, London NW1 2PG, UK; ^3^Department of Urology, Whipps Cross University Hospital, Barts Health NHS Trust, London E11 1SR, UK; ^4^Department of Cellular Pathology, University College London Hospitals NHS Foundation Trust, 4th Floor, Rockefeller Building, University Street, London WC1 6JJ, UK; ^5^Division of Surgery & Interventional Science, University College London, London WC1E 6BT, UK

## Abstract

Aggressive angiomyxoma is a rare mesenchymal tumour predominantly affecting the female pelvis and perineum but has also been described in males. This tumour can often present a diagnostic challenge and has a propensity for local recurrence after surgical excision. We present an unusual case of aggressive angiomyxoma arising from the bladder of a female patient which required local excision and Mitrofanoff formation.

## 1. Description of Case

**Figure 1:**
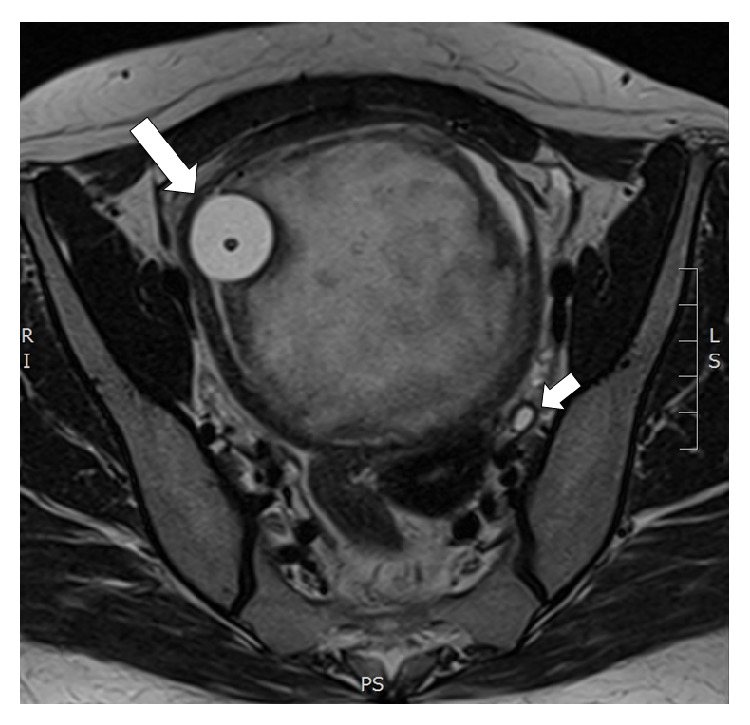
Axial section of T2 weighted MRI pelvis demonstrating solid mass within the bladder displacing the catheter balloon (large arrow) and causing bilateral hydronephrosis (small arrows).

A 48-year-old lady presented with visible haematuria and UTI. Pelvic examination revealed a mobile pelvic mass attached to the bladder neck. MRI revealed an 85 × 40 mm mass arising from the bladder neck. The tumour appeared hypointense on T1-sequence and had a swirled, heterogeneous appearance on contrast enhanced T2-sequences (Figure 1). Differential diagnoses included urethral diverticulum or leiomyoma.

The patient presented subsequently with acute kidney injury requiring bilateral percutaneous nephrostomy insertion. Examination under anaesthesia and cystoscopy with loop resection biopsies were performed. Histological analysis demonstrated fibromuscular tissue with moderately cellular lesions consisting of short spindle cells with intervening thick-walled ectatic blood vessels. There was focal evidence of myogenic differentiation but no significant cytological atypia, nor mitotic activity or necrosis. The proliferation fraction with MIB1 was 1%. The working diagnosis was low grade muscle sarcoma.

Surgical resection of the mass was undertaken via Pfannenstiel and vaginal approaches to gain access to the point of attachment of the mass in the left paraurethral area. Ultimately a urethrectomy was required, with concomitant bladder neck closure, Martius fat pad interposition, and creation of a catheterisable Mitrofanoff channel from the appendix. Nephrostomies were left in situ. Intraoperatively bilateral JJ stents were placed to protect the ureteric orifices during bladder neck closure.

**Figure 2:**
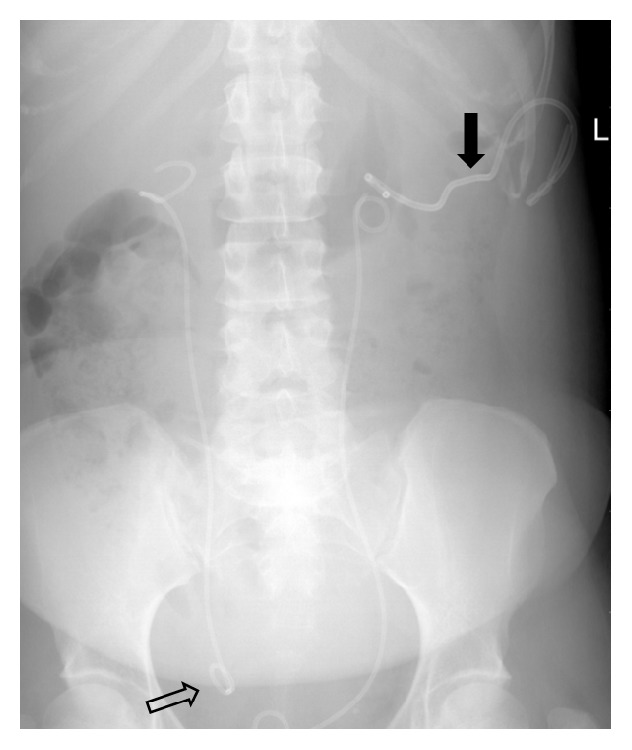
X-ray KUB following failed removal of encrusted left sided percutaneous nephrostomy tube (solid arrow) and right sided ureteric stent, which is displaced with the distal end lying in the distal ureter (hollow arrow).

The patient was discharged from hospital on day 15. At week three during fluoroscopic right nephrostomy removal, the distal J of the stent retracted into the distal ureter, and calcification of the left tube prevented its removal (Figure 2). At 12 weeks of bilateral PCNL, removal of left nephrostomy and both JJ stents was undertaken.

**Figure 3:**
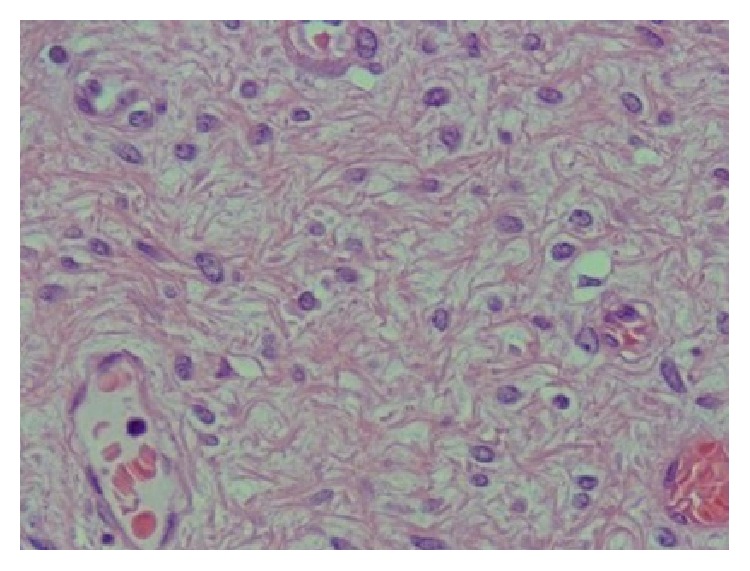
Colour slide of AAM from patient.

The mass measured 140 × 170 × 70 mm, with histopathological features of aggressive angiomyxoma (AAM) with spindle and epithelioid cells on fibrous stroma with a myxoid area (Figure 3). Although the mass was completely excised, the potential for local recurrence was noted and surveillance with periodic MRI scans of the pelvis is planned.

## 2. Discussion

AAM is a rare mesenchymal neoplastic tumour—the term “aggressive” was used to denote its propensity for local recurrence [1]. These neoplasms primarily arise in the pelvic and perineal region of adult females but have been described in males [2]. AAM can present a diagnostic challenge with nonspecific symptoms, leading to misdiagnosis with more common entities. AAM has a variable appearance on CT. On MRI, it is hypointense on T1-weighted images and hyperintense on T2-weighted images and demonstrates a characteristic swirling pattern with contrast enhancement, due to its myxoid nature [3].

Surgical excision remains the first-line treatment, although positive resection margins occur due to soft tissue infiltration and absence of a capsule [4]. However, positive surgical margins may not be prognostic: a review of 111 cases demonstrated no significant difference in time to recurrence with margin status [2]. Recurrence rates range 25–47%, usually within 5 years, and cases of metastasis have been reported; hence radiological follow-up is advocated [2, 5]. Though rare, clinical suspicion is required in order to not miss this diagnosis, and multidisciplinary care is key to obtaining a good outcome.
